# Regional differences of physical activity and sedentary behaviour in Swiss children are not explained by socio-demographics or the built environment

**DOI:** 10.1007/s00038-014-0645-8

**Published:** 2015-01-08

**Authors:** Bettina Bringolf-Isler, Urs Mäder, Alain Dössegger, Heidi Hofmann, Jardena J. Puder, Charlotte Braun-Fahrländer, Susi Kriemler

**Affiliations:** 1 Swiss Tropical and Public Health Institute, Socinstrasse 57, P.O. Box 4002, Basel, Switzerland; 2 University of Basel, Basel, Switzerland; 3 Swiss Federal Institute of Sport, Magglingen, Switzerland; 4 Center for Development and Environment, University of Bern, Bern, Switzerland; 5 University of Lausanne, Lausanne, Switzerland; 6 Institute of Social and Preventive Medicine, University of Zürich, Zurich, Switzerland

**Keywords:** Accelerometer, Childhood physical activity, Sedentary behaviour, Socio-cultural differences, Built environment, Socioeconomic neighbourhood

## Abstract

**Objective:**

We evaluated whether regional differences in physical activity (PA) and sedentary behaviour (SB) existed along language boundaries within Switzerland and whether potential differences would be explained by socio-demographics or environmental characteristics.

**Methods:**

We combined data of 611 children aged 4 to 7 years from four regional studies. PA and SB were assessed by accelerometers. Information about the socio-demographic background was obtained by questionnaires. Objective neighbourhood attributes could be linked to home addresses. Multivariate regression models were used to test associations between PA and SB and socio-demographic characteristics and neighbourhood attributes.

**Results:**

Children from the German compared to the French-speaking region were more physically active and less sedentary (by 10–15 %, *p* < 0.01). Although German-speaking children lived in a more favourable environment and a higher socioeconomic neighbourhood (differences *p* < 0.001), these characteristics did not explain the differences in PA behaviour between French and German speaking.

**Conclusions:**

Factors related to the language region, which might be culturally rooted were among the strongest correlates of PA and SB among Swiss children, independent of individual, social and environmental factors.

## Introduction

Physical inactivity is associated with several health outcomes like higher body weight (Must and Tybor 2005) features of the metabolic risk syndrome (Brage et al. 2004) and cardiovascular risk factors, even independently of body weight (Andersen et al. 2008). Sedentary behaviour (SB) is distinct from physical activity (PA) and affects health independently (Salmon et al. 2008). PA and low SB in children are therefore both acknowledged as important goals of prevention. A broad range of correlates of PA and SB in children that may guide public health strategies has been investigated (De Craemer et al. 2012). These included biological, demographic, psychological, socio-cultural and environmental factors. Gender, age, as well as influences by parents and peers have consistently been found to be associated with PA, but associations with environmental factors are less consistent. Whereas neighbourhood greenness was positively associated with PA in some studies (Grigsby-Toussaint et al. 2011; Roemmich et al. 2006), no such association was found in others (Lovasi et al. 2011). Positive associations with PA were also found for housing density (Roemmich et al. 2006) and land use mix (Lovasi et al. 2011). For the Swiss context, a recently published study found that more green was significantly associated with PA and SB in 4–10-year-old children (Bringolf-Isler et al. 2014). The same study suggested that the physical environment may interact with the socioeconomic neighbourhood.

Pronounced differences in self-reported PA and SB in children and young adults have been found in studies which examined regional influences across countries (Guthold et al. 2010). Yet, results from studies using objective measures such as accelerometers, which more reliably measure PA and SB in children (Chinapaw et al. 2010), were mixed (Riddoch et al. 2004; Verloigne et al. 2012) and differences existed even within countries (Burgi et al. 2010). To our knowledge for preschool children, no cross-country comparisons in objectively PA exist but in a pooled analysis of preschool children country differences in weight status were found (van Stralen et al. 2012). Regional differences in PA may be due to varying social environments, culturally related health beliefs, social capital, diverging built environments within which individual behaviour occurs, or differing sports and PA policies. As cross-country comparisons are hardly able to disentangle these factors, the present study focussed on regional differences between language boundaries in PA and SB levels within a single country. Switzerland is unique in encompassing different language and cultural regions within the same national political and administrative framework.

The present study aimed to evaluate (a) whether regional differences in PA and SB among children existed along the French/German language boundaries in Switzerland and whether (b) potential differences could be explained by differing patterns of personal, social or environmental characteristics.

## Methods

### Study population and setting

Four regional studies (Bringolf-Isler et al. 2009; Gehring et al. 2008; Niederer et al. 2009; Zahner et al. 2006) conducted between 2005 and 2009 among 4- to 7-year-old Swiss children using Actigraph accelerometers to assess PA have been combined into a single database. All data derived from observational studies (Bringolf-Isler et al. 2009; Gehring et al. 2008) or from baseline measures of intervention studies (Niederer et al. 2009; Zahner et al. 2006), and the selection of the respondents was comparable. Two studies included both children from the German and the French-speaking part of Switzerland (Bringolf-Isler et al. 2009; Niederer et al. 2009) and two included only children from the German-speaking part (Gehring et al. 2008; Zahner et al. 2006). To be included into this data pool raw accelerometer data, demographic information and an exact home address had to be available. A total of 800 children provided such data. Personal and social characteristics were obtained from parental questionnaires. The individual studies had been approved by the respective regional ethic committees (ethical committees of Aargau, Basel, Bern and St. Gallen). All participants or their parents gave written consent or assent for their participation.

### Personal characteristics

Information on personal characteristics was taken from the questionnaires. Low parental education was defined as none of the parents having compulsory education without further professional training and was contrasted to medium (2–4 year of apprenticeship) and high education (college or university). Nationality was subdivided into Swiss and non-Swiss (i.e. at least one parent with a non-Swiss nationality), language region distinguished between the German- and French-speaking part of the country, and number of siblings was divided into none, one, or two or more siblings. Mother’s employment was dichotomized into having an external job versus being a housewife. Height and weight were measured at school in three studies (Gehring et al. 2008; Niederer et al. 2009; Zahner et al. 2006) and reported by parental questionnaires in one study (Bringolf-Isler et al. 2009). In 45 children of the latter study, height and weight were assessed by parental questionnaire and simultaneously measured at school. A high correlation between reported and measured weight and height (both *r* = 0.95) and small mean deviances (−0.07 kg and −0.8 cm) was observed, allowing to combine the data. Overweight was defined according to the International Obesity Task Force (Cole et al. 2000). Season of data collection was defined as spring/summer (April to September) or autumn/winter (October to March).

### Accelerometer measures

All studies included used Actigraph accelerometers (models AM7164, GT1M or GT3X, Shalimar, Florida, USA) to measure physical activity. AM7164 and GT1M measure uni-dimensional and GT3X three-dimensional accelerations of the body. Accelerations are converted into arbitrary units (i.e. counts) over a predefined time period (epoch time). As the epoch times varied from 15 to 60 s among the four studies, all measures were re-integrated to an epoch time of 60 s using the software ActiLife (ActiLife version 4.9). The data reduction was conducted using the software MeterPlus (MeterPlus, version 4.2). Non-wearing time was defined as a period of 20 min of consecutive zero counts. Children were instructed to wear the accelerometers tightly fixed around the waist. Except for one study (Zahner et al. 2006) the accelerometers were not worn during sleeping hours. All accelerometers were removed for water activities. Children were included in the study if they accumulated at least two weekdays and one weekend day with at least 10 h of wearing time. Age-dependant cut-offs (Freedson et al. 2005) were used to define moderate-to-vigorous physical activity (MVPA), while SB was defined as an intensity of less than 100 counts per minute (cpm) (Trost et al. 2011).

### Environmental factors

Environmental data could be linked to valid addresses through the geographic information system (GIS) ArcGis (ESRI 2011), census data (Bundesamt für Statistik 2009), and land use statistics (Bundesamt für Statistik 2009). All environmental data could be matched close to the time point of data collection. The selection of the environmental variables was based on previous analyses (Bringolf-Isler et al. 2014; Grasser et al. 2013). SwissTopo Vector25 (version 2008) calculated in ArcGIS10 (ESRI 2011. ArcGis Desktop: Release 10) was used to calculate the length of main street segments within 200 metres (m), around the place of residence (main street density) and the number of cul de sacs and intersections within 500 m around the place of residence. Information about population density, building density, mixed land use and the number of schoolchildren living within the neighbourhood was based on census data. These data were available per hectare (ha) and were summed up to 3 × 3 ha around the place of residence to correspond to a maximum buffer of 200 m. To compute a mixed land use, score buildings within a buffer of 200 m were categorized as residential, recreational, retail, office or institutional (Bringolf-Isler et al. 2014). A sum of each building category present in the buffer was calculated, resulting in a score ranging from 1 to 5. A score of 1 denotes residential only, while a score of 5 indicates a high land use mix. Information on green spaces (parks, play grounds and meadows) and wooded areas was based on land use statistics, which differentiate between 72 types of land use per hectare. The number of hectares of green spaces and of wooded areas was calculated for the near neighbourhood (3 × 3 ha).The characterization of the socioeconomic neighbourhood was based on the Swiss neighbourhood index of socioeconomic position (Swiss-SEP) (Panczak et al. 2012). The index is composed of the median rent per square metre, the proportion of households headed by a person with a primary education or less, the proportion of households headed by a person in manual or unskilled occupation and the mean number of persons per room. The neighbourhood boundaries for each building were defined by the road network connectivity resulting in one buffer per child. The Swiss-SEP score is divided into deciles, 1 representing the lowest socioeconomic decile and 10 the highest. Definitions and units of the neighbourhood attributes are presented in Table 2.

### Statistical analyses

Values are reported as means and standard deviations for continuous variables and percentages for categorical variables. Descriptive analyses of personal characteristics and neighbourhood attributes were performed for the whole sample and stratified by language region. Differences by regions were tested using Chi2- or Kruskal–Wallis-test.

Initially, a basic regression model (model 1) adjusted for sex, age, accelerometer type, season and study cluster (individual study included in the data pool) was used to test the respective associations between total PA (in cpm),  %-MVPA and  %-SB (defined as MVPA and sedentary behaviour per measuring time), and individual socio-demographic characteristics and neighbourhood attributes. As the distribution of the residuals was skewed, standard errors of the regression estimates were determined using a bootstrap with 1000 replications. Although measures of the vertical axis of AM7164, GT1M and GT3X devices are reported to be comparable in older youth and adults (Hanggi et al. 2013; Kozey et al. 2010), the accelerometer type was included as a covariate to exclude bias.

Second, to evaluate whether socio-demographic factors that were significantly associated with TPA  %-MVPA or  %-SB in model 1, or overweight would explain the association between language region and PA measures, these characteristics were additionally included in the regression models (model 2). Thirdly, environmental characteristics which differed between the two language regions were added to the models 2 resulting in the fully adjusted model 3.

In addition, interactions by language region of the association between TPA,  %-MVPA and  %-SB and socio-demographic and environmental factors were tested. All analyses were conducted using STATA 12.0.

## Results

Personal and social characteristics were available for 800 children. 650 children had valid accelerometer data, and 611 children (76.4 %) provided a valid home address, which allowed linkages to geo-coded objective environmental data. The mean age of included children was significantly higher (5.8 and 5.5 years, respectively) and significantly more children were included in the German-speaking part (81 and 70 %, respectively). No difference was found by sex. The final study population consisted of 395 (64.6 %) children from the German-speaking part and 216 (35.4 %) from the French-speaking part of Switzerland (Table 1). French-speaking children were significantly younger, had more often less educated parents and were of non-Swiss nationality (*p* < 0.001). They had fewer siblings (*p* < 0.01), and were less physically active and more sedentary than children from the German-speaking part of Switzerland (*p* < 0.001) No difference by language region was found for parental education, employment of the mother and overweight.

**Table 1:** Characteristics of the study population

		Total (*n* = 611)	Region		*p*-value
			German (*n* = 395)	French (*n* = 216)	
Age Years		5.7 (1.0)	6.2 (0.8)	5.0 (0.8)	≤0.001
Sex, *n* (%) Girls Boys		299 (48.9)	188 (47.6)	111 (51.4)	
		312 (51.1)	207 (52.4)	105 (48.6)	0.4
Parental education, *n* (%) Low Medium High		64 (11.1)	27 (7.3)	37 (17.8)	
		257 (44.4)	192 (51.7)	65 (31.3)	≤0.001
		258 (44.5)	152 (58.9)	106 (51.0)	
Nationality, *n* (%) Swiss Non-Swiss		413 (67.6 %)	305 (77.2)	108 (50.0)	
		198 (32.4 %)	90 (22.8)	108 (50.0)	≤0.001
Number of siblings, *n* (%) None One Two and more		126 (20.6)	74 (18.7)	52 (24.1)	
		430 (70.4)	276 (69.9)	154 (71.3)	
		55 (9.0)	45 (11.4)	10 (4.6)	≤0.01
Employment mother, *n* (%) Yes No (housewife)		248 (46.4)	125 (36.8)	123 (63.1)	
		287 (53.6)	215 (63.2)	72 (36.9)	0.1
Overweight, *n* (%) No Yes		540 (90.3)	350 (91.2)	190 (88.8)	
		58 (9.7)	34 (8.9)	24 (11.2)	0.4
Data collection, *n* (%) Summer Winter		382 (62.5)	276 (69.9)	106 (49.1)	
		229 (37.5)	119 (30.1)	110 (50.9)	≤0.01
Total PA^a^ Counts per min		677.5 (176.6)	716 (9.59)	606.5 (14.2)	<0.001
% MVPA^a^ MVPA/total wearing time		21.3 (4.4)	22.2 (0.24)	19.6 (0.35)	<0.001
% Sedentary^a^ Sedentary time/total wearing time		24.90 (6.2)	22.8 (0.32)	26.2 (0.47)	<0.001

Switzerland 2005–2010 (KISS, SCARPOL. Ballabeina and Pasture). Values are means (SD) or numbers and percentages [*n* (%)]

^a^Adjusted for sex, age, study cluster and accelerometer type

Many neighbourhood attributes at the children’s place of residence differed statistically significantly between the French- and the German-speaking part of Switzerland (Table 2). In the French-speaking part, main street density, population density, the mixed land use score (*p* < 0.001) and the schoolchildren density (*p* = 0.04) were higher than in the German-speaking part, whereas the building density, the number of cul de sac streets, the amount of green spaces and wooded areas and the Swiss socioeconomic neighbourhood score were significantly lower (*p* < 0.001).

**Table 2:** Distribution of GIS and census-based environmental factors by language region within Switzerland, 2005–2010 (KISS, SCARPOL. Ballabeina and Pasture)

		Total (*n* = 611)	Region		*p*-value
			German (*n* = 395)	French (*n* = 216)	
Main streets * m* in a buffer of 200 m		488.4 (370.5)	368.8 (329.6)	707.0 (340.6)	<0.001
Population density * n* × 9 ha^−1^		533.2 (428.4)	369.5 (309.9)	832.5 (453.2)	<0.001
Building density * n* × 9 ha^−1^		53.8 (32.4)	58.1 (35.9)	46.0 (23.0)	<0.001
Mixed land use Score (1 to 5)^a^		2.9 (1.7)	2.6 (1.7)	3.5 (1.7)	<0.001
Intersections Number of intersections (three or more streets) in a buffer of 500 m		56.8 (24.7)	57.9 (28.9)	54.8 (14.2)	0.96
Cul de sac streets Number in a buffer of 500 m		7.0 (4.1)	7.6 (4.4)	6.0 (3.5)	<0.001
Green playing areas Number of assigned ha × 9 ha^−1^		1.1 (1.7)	1.5 (2.0)	0.3 (0.6)	<0.001
Woods Number of assigned ha × ha^−1^		0.6 (1.1)	0.7 (1.2)	0.3 (0.8)	<0.001
School children density * n* × 49 ha^−1^		15.4 (17.7)	13.4 (12.6)	19.0 (24.1)	0.004
Swiss socioeconomic neighbourhood-score^b^ Score (1 to 10)		5.1 (2.9)	5.7 (2.8)	3.9 (2.7)	<0.001

^a^Score range of 1 to 5, indicating the presence or the absence of five types of land use (residential, entertainment, retail, office, institutional) per 9 ha

^b^Deciles of neighbourhoods (1 = low SES) based on the median rent per square metre, the proportion of household headed by a person with primary education or less, the proportion headed by a person with manual or unskilled occupation and the mean number of persons per room

Table 3 shows the respective associations between TPA,  %-MVPA,  %-SB and the socio-demographic characteristics and the neighbourhood attributes adjusted for the basic characteristics sex, age, accelerometer type, season and study cluster (model 1). Boys were more active than girls, and children were more active and less sedentary in summer than in winter, whereas age was neither significantly associated with PA nor with SB. Children from the French-speaking part showed 13 % less TPA, 11 % less  %-MVPA and 13 % higher  %-SB (i.e. 13 % more minutes of  %-SB per day) than those from the German-speaking part. Non-Swiss children and children living in a neighbourhood with higher main street density were less physically active, whereas children living in a higher socioeconomic neighbourhood were more physically active compared to their respective counterparts. All other tested socio-demographic and environmental factors showed no significant association with PA or SB.

**Table 3:** Association of socio-demographic and neighbourhood factors with physical activity and sedentary behaviour (adjusted for sex, age, accelerometer type and season of the measurement, study and wearing time); *n* = 1742)

		TPA [*β*-coeff (95 % CI)]	MVPA [*β*-coeff (95 % CI)]	SB [*β*-coeff (95 % CI)]
Socio-cultural factors				
Swiss language region German French		0 (Reference group)		
		−90.4 (−128.0 to −52.8)***	−2.4 (−3.3 to −1.4)***	3.3 (2.2 to 4.5)***
Highest parental education Low Medium High		0 (Reference group)		
		−30.3 (−82.8 to 22.3)	−0.8 (−2.0 to 0.5)	0.9 (−0.7 to 2.4)
		−22.7 (72.1 to 26.7)	−0.5 (−1.6 to 0.6)	1.1 (−0.5 to 2.6)
Nationality Swiss Non-Swiss		0 (Reference group)		
		−42.0 (−70.5 to −13.6)**	−1.1 (−1.8 to −0.3)**	0.2 (−0.9 to 1.2)
Number of siblings None One Two and more		0 (Reference group)		
		1.6 (−35.2 to 38.5)	0.2 (−0.6 to 1.1)	−0.2 (−1.5 to 1.0)
		7.5 (−33.6 to 48.5)	0.2 (−0.8 to 1.2)*	−0.1 (−1.4 to 1.3)
Employment of mother External job Housewife		0 (Reference group)		
		24.7 (−7.8 to 57.3)	0.5 (−0.3 to 1.3)	−0.5 (−1.6 to 0.6)
Season of data collection^a^ Summer Winter		0 (Reference group)		
		−72.3 (−101.2 to −43.3)***	−1.4 (−2.2 to −0.6)***	0.9 (−0.1 to 2.0)
Personal factors				
Age^a^ Increase per year		−2.5 (−27.8 to 22.9)	−0.5 (−1.1 to 0.2)	0.2 (−0.7 to 1.0)
Sex^a^ Boys Girls		0 (Reference group)		
		−60.8 (−87.7 to 33.9)***	−1.5 (−2.2 to −0.9)***	0.7 (−0.1 to 1.6)
Overweight No Yes		0 (Reference group)		
		−16.4 (−54.8 to 22.0)	−0.3 (−1.3 to −0.8)	0.0 (−1.4 to 1.4)
Environmental factors				
Main streets Increase per IQR^b^		−24.0 (−42.2 to −5.7)**	−0.5 (−1.0 to −0.0)*	0.4 (−0.3 to 1.1)
Population density Increase per IQR^b^		−13.7 (−34.3 to 6.9)	−0.3 (−0.9 to 0.3)	0.2 (−0.6 to 1.0)
Building density Increase per IQR		10.3 (−8.6 to 29.2)	0.3 (−0.2 to 0.8)	−0.3 (−6.4 to 3.8)
Mixed land use Increase per IQR^b^		2.6 (−20.1 to 25.3)	0.0 (−0.5 to 0.6)	−0.0 (−0.7 to 0.7)
Green space Increase per IQR^b^		9.2 (−1.0 to 19.5)	0.2 (−0.1 to 0.4)	−0.2(−0.4 to 0.0)
Woods Increase per IQR^b^		4.6 (−7.1 to 16.4)	−0.0 (−0.3 to 0.3)	0.0 (−0.4 to 3.8)
Schoolchildren density Increase per IQR^b^		0.1 (−13.5 to 13.7)	−0.1 (−0.4 to 0.2)	0.2 (−0.1 to 0.6)
Swiss Socioeconomic neighbourhood index Increase per IQR^b^		32.8 (4.8 to 60.6)*	0.8 (0.1 to 1.5)*	−0.1 (−0.3 to 0.1)

Switzerland 2005–2010 (KISS, SCARPOL. Ballabeina and Pasture)

*TPA* total physical activity in cpm, *MVPA* moderate-to-vigorous physical activity, *SB* sedentary behaviour

* *p* ≤ 0.05; ** *p* < 0.01; *** *p* ≤ 0.001

^a^Not adjusted for itself

^b^Increase from the 25th to the 75th percentile (interquartile range, IQR) of each environmental attribute

**Fig. 1:**
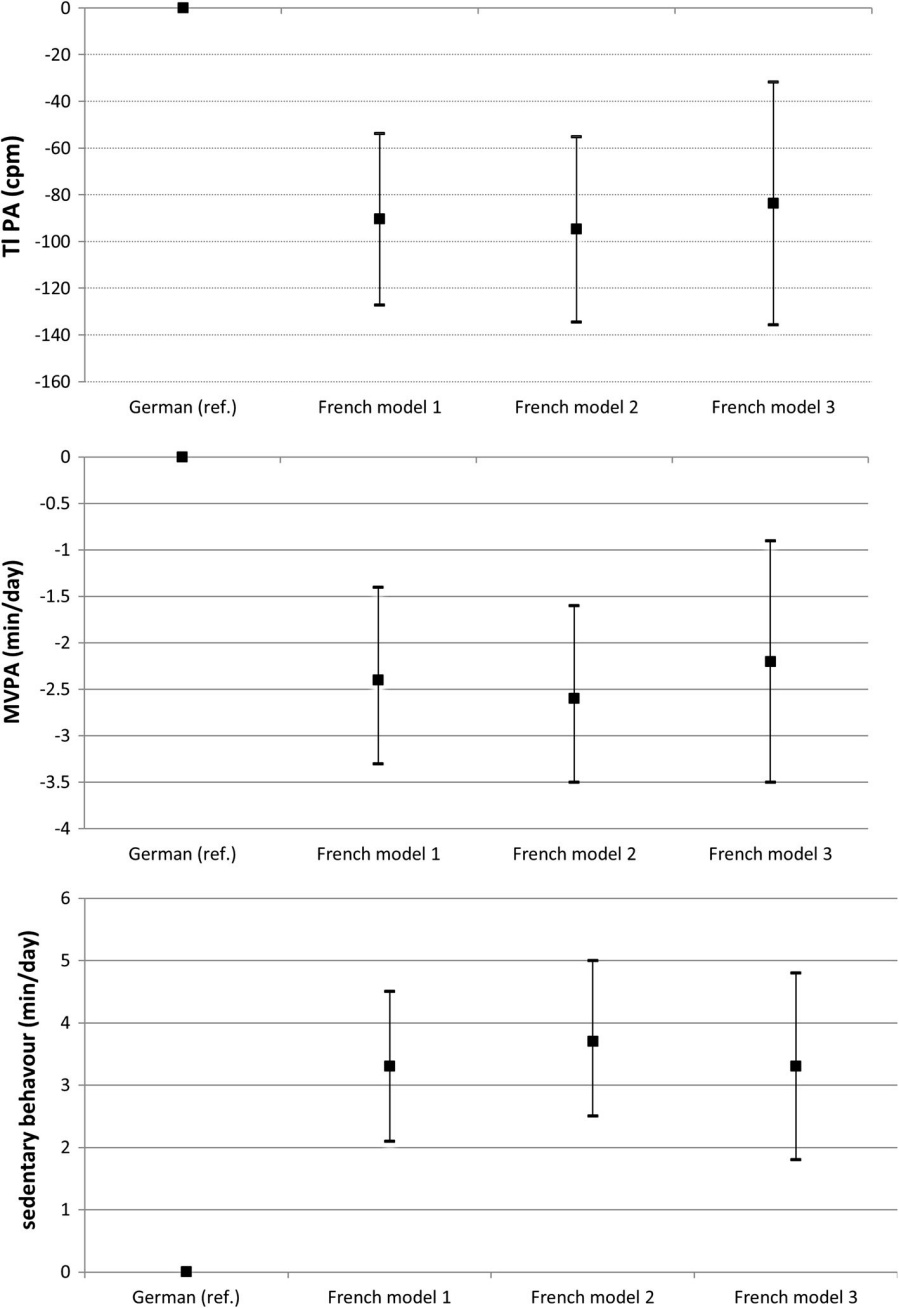
Differences in PA and SB between language regions according to three different adjustment models. Switzerland 2005–2010 (KISS, SCARPOL, Ballabeina and Pasture) The German-speaking region was taken as reference. The difference in total PA,  %-MVPA and  %-SB between the two language areas remained similar for the basic model 1 (adjusting for age, sex, study cluster, season of measurement and type of accelerometer), the model 2 with further adjustment for socio-demographic factors and overweight and the final model 3 with additional adjustments for environmental factors (difference between language regions: *p* < 0001 for all models)

The difference in TPA,  %-MVPA and  %-SB in children living in the French-speaking part compared to German-speaking part for model 1 (basic model), model 2 (after adjustment for socio-demographic factors and overweight) and model 3 (after additional adjustment for environmental factors) is displayed in Fig. 1. Estimates of TPA,  %-MVPA and  %-SB in the basic model 1 did not substantially change after additional adjustments for social factors and overweight (13, 12, 14 %, model 2), nor after the inclusion of neighbourhood attributes (12, 10, 13 %, model 3). The explained variability of the model increased slightly from *R* ^2^ = 0.13, 0.11 and 0.22 for TPA,  %-MVPA and  %-SB, respectively, for models 1 to *R* ^2^ = 0.17, 0.16 and 0.24, respectively, for models 3.

When effect modification of the association between PA measures and socio-demographic and environmental variables by language region was tested, significant interactions were found. Whereas the number of cul de sac streets was positively associated with TPA and  %-MVPA in children from the French-speaking part, a negative association was observed in children from the German-speaking part (*p* value of the likelihood ratio test = 0.04 and 0.03 for TPA and  %-MVPA, respectively). Moreover, main street density increased  %-SB more strongly in French as compared to German-speaking children (likelihood ratio test *p* = 0.03), and building density was more strongly inversely related to  %-SB in French compared to German-speaking children (likelihood ratio test *p* = 0.02). An increase of green spaces in the neighbourhood tended to be more strongly associated with less sedentary time in German as compared to French-speaking children (likelihood ratio test: *p* = 0.01) and the Swiss-SEP tended to be inversely associated with SB in German-speaking children and positively in French-speaking children although not statistically significant in both regions (likelihood ration test: *p* = 0.05). For all other socio-demographic or environmental factors, no interaction with language region was statistically significant.

## Discussion

The present study highlights that pronounced differences in PA and SB are observed between children living in two distinct language regions within a single country with similar national health policies and regulations. Although the two regions differed significantly with respect to several environmental characteristics and a number of socio-demographic factors, those characteristics did not explain the regional differences in PA and SB. These results underline that other aspects which differ by language region may play an important role in determining PA and SB in children including behavioural differences as health attitudes and perceptions are culturally rooted.

Previous studies reporting variations in objectively measured PA and SB between countries (Ekelund et al. 2012; Verloigne et al. 2012) claimed that different sport policies and access to sport facilities might explain country differences (Verloigne et al. 2012). In the present Swiss study, however, regional differences in PA and SB were noted although sport policies such as the number of physical education classes at school and the support for sport programmes are the same in all language regions, thus—at least for young children—precluding differing sports policies as an explanation.

A previous study in Swiss preschool children (Burgi et al. 2010) reported similar differences in PA behaviour along the language boundaries. However, that study could not exclude that characteristics of the built environmental might explain the regional differences. The neighbourhood environment has been shown to influence physical activity in adults and children likewise (Bringolf-Isler et al. 2014; Grasser et al. 2013). However, in the present study, aspects of the built environment did not explain the regional differences although several of these factors were related to PA. Language region was even a stronger correlate of PA and SB than migration background or educational level of the parents, factors that have been found to influence PA or SB in previous studies (De Craemer et al. 2012; Hesketh et al. 2006).

The tested socio-demographic and environmental factors did not explain the differences in PA and SB found by language regions bringing up the influence of the cultural background and of the children. Cultural factors explained a significant proportion of the variance in safety concerns in a study testing PA-related parenting practices (O’Connor et al. 2014). Moreover, objectively assessed parental PA has been shown to be associated with PA in their children at ages 5–6 years (Jago et al. 2014) stressing the possible influence of parental behaviour, their cultural background and their health attitudes on the behaviour of their offspring’s irrespective of policies, socio-demographics and the built environment. Previous studies showed that behavioural differences according to language boundaries can even exist within a single town: A study in the bilingual city of Biel/Bienne in Switzerland illustrated that French primary school children spent significantly less time playing vigorously outdoors than their German-speaking peers (Bringolf-Isler et al. 2010), and parents of French-speaking children were significantly more likely to drive their children to school compared to parents of German-speaking children, although distance to school, the level of urbanization and other environmental characteristics were comparable (Bringolf-Isler et al. 2008). Similarly, among Belgium adults, differences in utilitarian cycling were found between adults living in Flanders as compared to Brussels or Wallonia which were independent of environmental correlates and are likely to be rooted in the Flemish culture (de Geus et al. 2014). Interestingly, differences in health relevant behaviour between language regions in Switzerland are not limited to measures of PA. Among Swiss adults, more smoking and alcohol consumption have been reported by French compared to German-speaking persons, and this went along with differentials in cause-specific mortality despite similar all-cause mortality (Faeh et al. 2009). Health behaviour and health attitudes in a single country thus do not have to be the same for the whole population, if different cultures and language regions exist (Humpel et al. 2002). PA and SB may be influenced by other social factors not assessed here such as friends’ and parents’ support, encouragement, participation, perceptions and other mechanisms. Moreover, it has been shown that besides health risk behaviours also their correlates can differ by country (Behanova et al. 2014) pointing to an effect modification by culture. All these aspects have to be understood to plan appropriate tailored interventions.

Strength of the present study is the relatively large number of objective measurements of PA in young children collected in a single country using standardized procedures for data collection and analysis. However, there are also noteworthy limitations. The assessment of the environment was limited to available objective information, which could be linked to the home address. Yet, parents’ subjective perceptions of the environment, their fears and safety concerns or indicators of social coherence and social capital were not available for analysis. Such subjective attitudes might, however, influence children’s use of the physical environment and may also be influenced by cultural factors. Thus, future studies should include both the objective environment and the subjective assessment of the environment. Nevertheless, evaluating the impact of objective environmental factors on PA and SB may improve our understanding of modifiable factors for PA, which could be translated into public health strategies to increase PA and reduce SB (Ding et al. 2013). The combination of four studies can bring interesting findings, but it causes also methodological problems. Although we controlled for study cluster and accelerometer device, we cannot completely exclude that the heterogeneity of the data collection affected the result. In addition, epoch time had to be re-integrated to 60 s. As a consequence, the prolongation of the epoch time can slightly shorten the classification of MVPA and prolong time spent with SB (Ojiambo et al. 2011). Accelerometers are considered valid and reliable measurement devices but they have some limitations, too. This includes the inability to assess water activities and to properly assess PA of upper body training, or gliding activities without accelerations of the body (Corder et al. 2008). Nevertheless, one can expect that these differences in activity probably differ in a manner similar to the measured activities and that neither upper body training nor swim lessons have a big impact on PA in children aged 4 to 7. Furthermore, the short measurement period of at least 3 days limits the generalizability of the findings. However, 90 % of the children provided more than 3 valid days and a sensitivity analysis excluding the children with a short measurement did not change any result. A further limitation is the cross-sectional nature of the study precluding any suggestion of causality.

### Conclusion

The language region carried out its own independent influence on PA and SB in Swiss children, independent of individual, social and environmental factors. These differences might be culturally rooted but not understood so far. Cultural differences have to be taken into account when transferring public health strategies and interventions. They should be adapted to socio-cultural norms, even within the same country. To promote and support healthy behaviour of the population, and to better understand cross-country differences, knowledge about culturally rooted health beliefs and behaviour is needed.
